# Fabrication of NiO/NiCo_2_O_4_ Mixtures as Excellent Microwave Absorbers

**DOI:** 10.1186/s11671-019-2988-9

**Published:** 2019-05-07

**Authors:** Xiankun Cheng, Xiangbo Zhou, Shipeng Wang, Zhongliang Liu, Qinzhuang Liu, Yongxing Zhang, Qiangchun Liu, Bing Li

**Affiliations:** School of Physics and Electronic Information, Huaibei Normal University, Huaibei, 235000 People’s Republic of China

**Keywords:** NiO/NiCo_2_O_4_, Mixtures, Yolk-shell, Electromagnetic wave absorption

## Abstract

The NiO/NiCo_2_O_4_ mixtures with unique yolk-shell structure were synthesized by a simple hydrothermal route and subsequent thermal treatment. The elemental distribution, composition, and microstructure of the samples were characterized by transmission electron microscopy (TEM), X-ray diffraction (XRD), and scanning electron microscope (SEM), respectively. The microwave absorption property was investigated by using vector network analysis (VNA). The results indicated that the excellent electromagnetic wave absorption property of the NiO/NiCo_2_O_4_ mixtures was achieved due to the unique yolk-shell structure. In detail, the maximum reflection loss (RL) value of the sample reached up to − 37.0 dB at 12.2 GHz and the absorption bandwidth with RL below − 10 dB was 4.0 GHz with a 2.0-mm-thick absorber. In addition, the NiO/NiCo_2_O_4_ mixtures prepared at high temperature, exhibited excellent thermal stability. Possible mechanisms were investigated for improving the microwave absorption properties of the samples.

**Electronic supplementary material:**

The online version of this article (10.1186/s11671-019-2988-9) contains supplementary material, which is available to authorized users.

## Background

Recently, with arising and development of wireless communication and the wide application of electronic devices, electromagnetic contamination has become serious problems to the electronic equipment [1]. High-power electromagnetic wave in regional environment can interfere with each other, which can result in the damage of communication system or even cause serious accidents, such as missile error, aircraft crash, and other disastrous consequences. Therefore, to develop a high-efficiency electromagnetic wave absorption (EMW) absorber with strong absorption, wide bandwidth, small thickness, and lightweight is highly desirable.

Currently, the study on EMW absorbers is mainly concentrated on transition-metal oxides [2, 3], binary metal oxides [4], carbonaceous materials [5–7], conducting polymers [8], magnetic materials [9–12], Metal–organic-frameworks materials [13, 14], and graphene-based hybrid materials [15–21]. At present, NiO and NiCo_2_O_4_ have attracted tremendous interest due to its unique properties in the absorption intensity and frequency bandwidth of electromagnetic wave. As we all know, NiCo_2_O_4_ is a hybrid transition metal oxide with excellent electrical and electrochemical properties [22, 23] The potential of NiCo_2_O_4_ [24] and NiCo_2_O_4_@PVDF composite [25] for electromagnetic wave absorption has been studied. Interestingly, recent research also demonstrated the potential of NiO and its related mixtures in the application of microwave absorption [26, 27]. Therefore, the combination of NiO and NiCo_2_O_4_ for the preparation of electromagnetic wave absorbing materials has become a new research field. For instance, Liu et al. [28] conducted some explorations on the EMW absorption properties of NiCo_2_O_4_/Co_3_O_4_/NiO composites. Their results demonstrated that the sample exhibited a maximum RL value of − 28.6 dB at 14.96 GHz. Porous NiO/NiCo_2_O_4_ lotus root-like nanoflakes were demonstrated by Liang and co-workers as a promising candidate for a microwave absorbent [29]. The NiO/NiCo_2_O_4_ (60 wt%)-wax hybrid showed the strongest EMW absorption with the RL value of − 47 dB at 13.4 GHz. However, the method used in the preparation of the NiO/NiCo_2_O_4_ hybrid process is too complicated to be suitable for mass production. As a result, the development of a facile method for the preparation yolk-shell NiO/NiCO_2_O_4_ mixtures with excellent EMW performance is still an intriguing topic.

Herein, we report a simple hydrothermal method and subsequent post-thermal treatment to prepare the NiO/NiCo_2_O_4_ mixtures with unique yolk-shell structure. Results indicated that the obtained samples exhibited excellent microwave absorbing performance. The relationship between structure, surface morphologies, and the microwave absorbing performance was also discussed. The current study will greatly expand the application scenario of the NiO/NiCo_2_O_4_ mixtures as electromagnetic wave absorber.

## Methods

The precursor was first prepared using a simple hydrothermal method. In a typical synthesis, 1 mmol of Ni(NO_3_)_2_, 2 mmol of Co(NO_3_)_2_·6H_2_O, and 0.6 mol of urea (H_2_NCONH_2_) were dissolved in 5 mL isopropanol (C_3_H_8_O) and 25 mL of deionized water and then stirred for 0.5 h to make them full dispersed. Then, the resultant solution was transferred to a polytetrafluoroethylene reactor and reacted at 120 °C for 12 h. After that, the autoclave was cooled down to room temperature naturally. Then, the sample was collected by centrifuge and washed several times with alcohol and deionized water, respectively. The obtained wet powder was dried at 60 °C for 10 h in a vacuum oven. Pink precipitates were further calcined at 350 °C, 450 °C, 550 °C, and 650 °C for 3.5 h under atmospheric conditions, respectively. The reagents used in the assay were all analytically pure and used without further purification.

The crystalline phases of the calcined products were characterized by X-ray diffractometer (XRD, PANalytical, Empyrean) using Cu *Kα* radiation (λ = 1.54178 Å, 40.0 kV). Structures, morphologies, composition, and elemental distribution of the samples were observed by using scanning electron microscopy (SEM, JEOL-6610LV) and transmission electron microscopy (TEM, JEM-2100, INCAX-Max80). The electromagnetic parameters of the obtained samples were examined by the vector network analyzer (VNA, AV3629D) using transmission-reflection mode in the frequency range of 2.0–18.0 GHz at room temperature. The samples with different annealing temperatures (350 °C, 450 °C, 550 °C, 650 °C) are labeled as S1, S2, S3, and S4, respectively to the convenience of this description.

## Results and Discussion

**Fig. 1:**
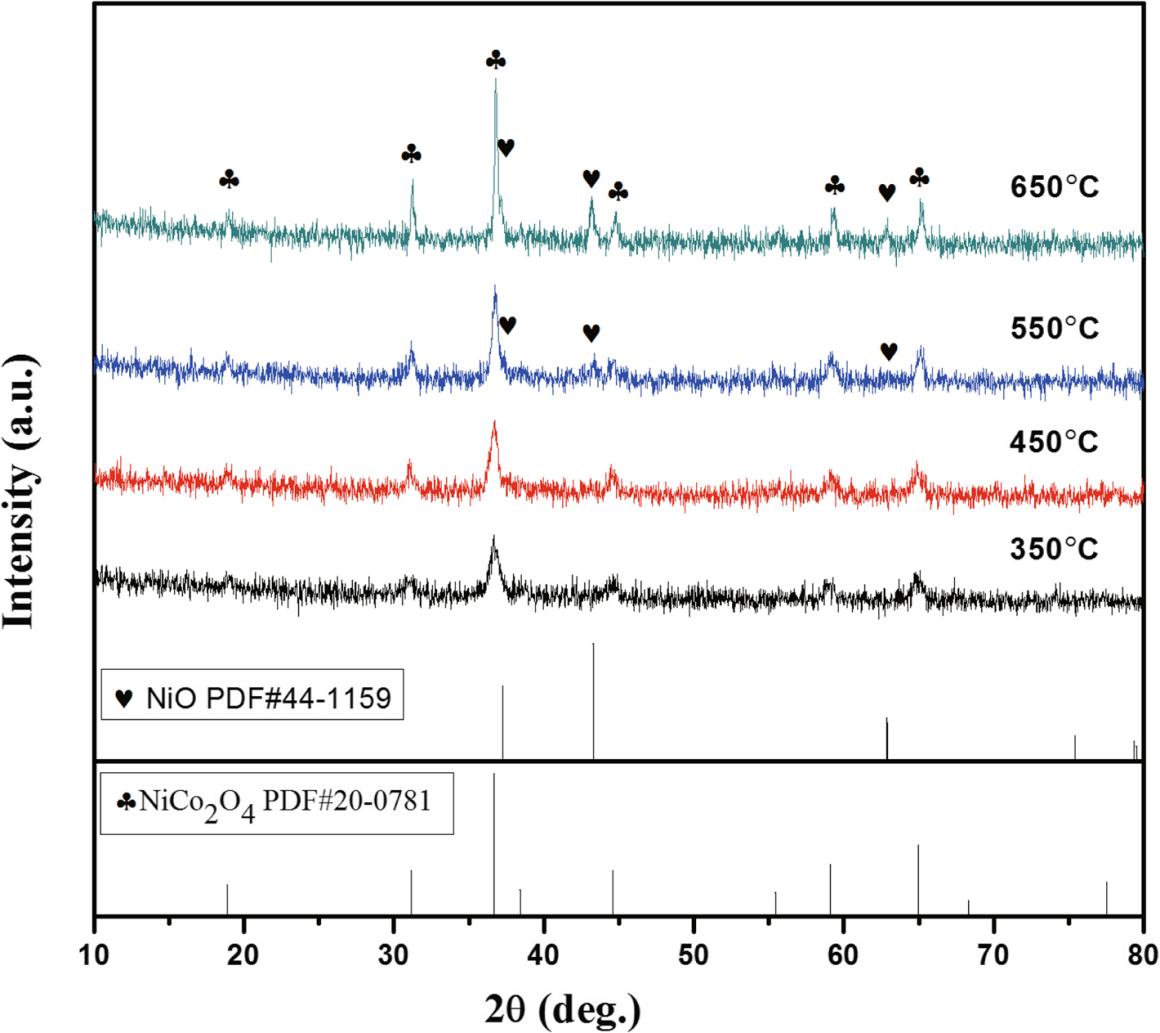
XRD patterns of the samples

The X-ray characteristic spectra of the samples at different annealing temperatures are shown in Fig. 1. Comparing the standard cards of NiO (PDF#44-1159) and NiCo_2_O_4_ (PDF#20-0781), it is found the diffraction peaks of the samples with annealing temperature of 650 °C and 550 °C correspond to NiO (2*θ* = 37.2°, 43.3°, and 62.9°) and NiCo_2_O_4_ (2*θ =* 31.1°, 36.7°, 44.6°, 59.1°, and 64.9°), respectively. The XRD patterns show that the NiO/NiCo_2_O_4_ mixtures are successfully synthesized by using the raw materials mentioned in the experiment. However, the diffraction peaks of NiO are not found in samples with annealing temperature less than 550 °C, indicating that high temperature is favorable for the formation of NiO. In the hydrothermal reaction stage, due to the participation of urea, we obtain a small amount of NiCO_3_, which can be decomposed to NiO and CO_2_ at high temperature. At the same time, with the increase of annealing temperature, the crystallinity of NiCo_2_O_4_ crystals is also optimized, which means that the samples can be used in a high-temperature environment.

**Fig. 2:**
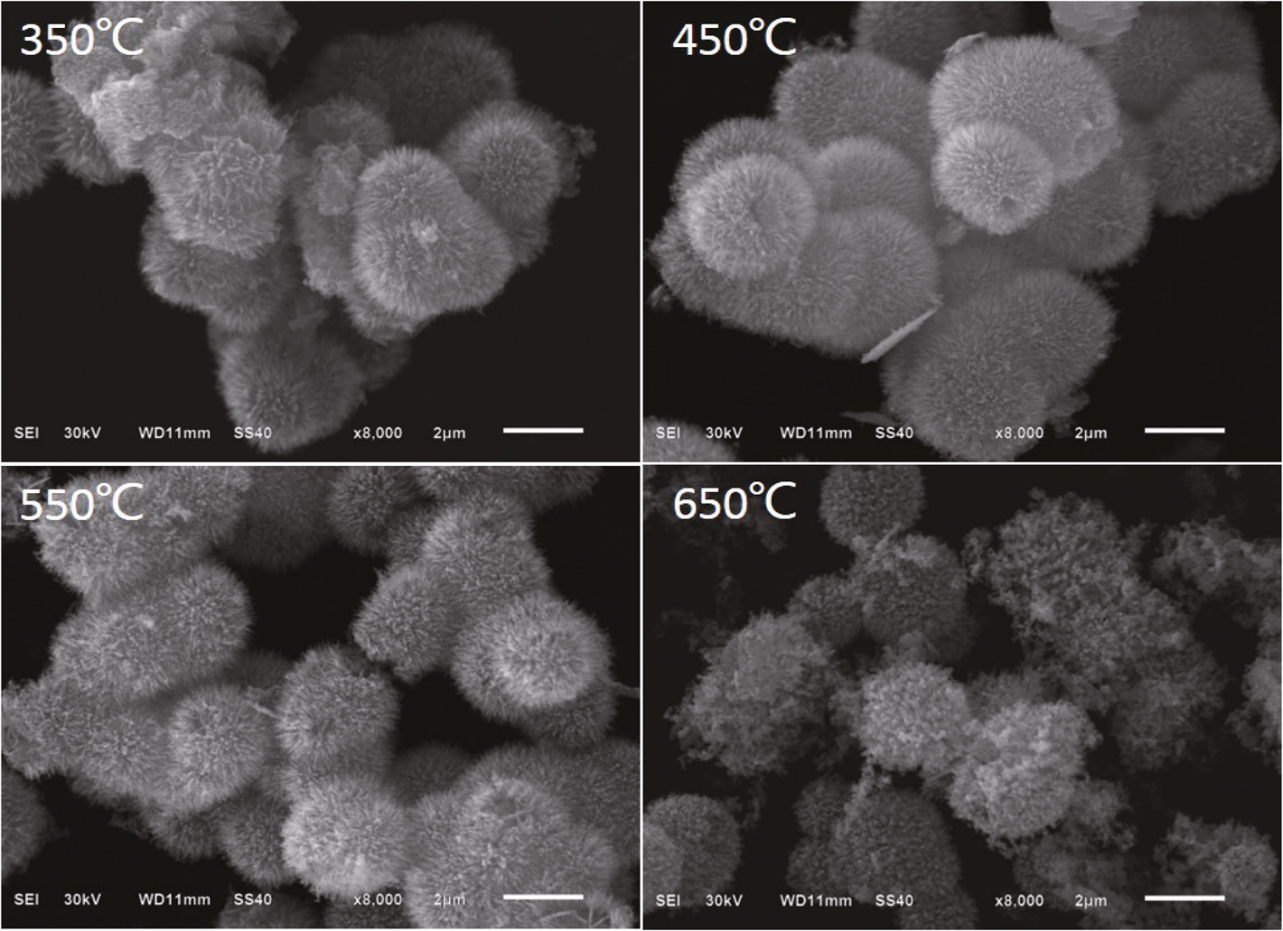
SEM images of NiCo_2_O_4_ particles and NiO/NiCo_2_O_4_ mixtures

SEM images of all samples are shown in Fig. 2. As shown in the micrographs of the sample, most of the samples exhibited microspheres of different diameters with a vast of radial nanowires on the surface. However, with the annealing temperature increasing, the surface of the sample cracks and a mass of pores are generated, such as the sample corresponding to the annealing temperature of 650 °C.

**Fig. 3:**
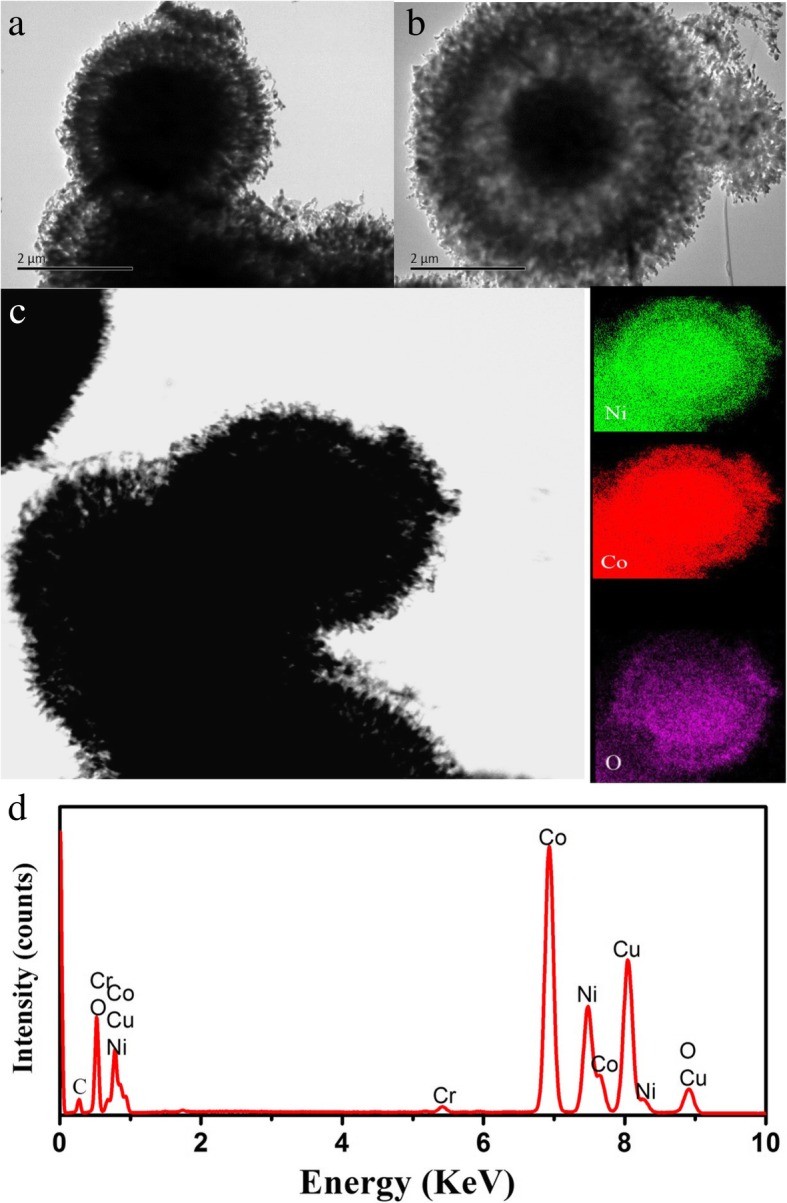
The TEM images (**a**–**c**) and EDS image (**d**) of NiO/NiCo_2_O_4_ mixtures with the annealing temperature of 650 °C

In order to further investigate the microstructure and the distribution of NiO and NiCo_2_O_4_ in the NiO/NiCo_2_O_4_ mixtures, transmission electron microscopy (TEM), and electron diffraction spectrum (EDS) was used to measure the sample with an annealing temperature of 650 °C. From Fig. 3a, b, we can see a typical yolk-shell structure. In Fig. 3c, Co elements are mainly concentrated on the kernel part. Therefore, it can be inferred that NiCo_2_O_4_ is mainly distributed within the nucleus. According to the distribution of Ni elements shown in Fig. 3d, there is a clear gap between the shell layer and the kernel part, which is somewhat different from the distribution of Co elements. Combining with the XRD patterns of the sample, it can be inferred that NiO is mainly distributed on the outer sphere of the whole hollow core-shell structure. The composition is verified by EDS spectroscopy as shown in Fig. 3d [30]. In addition, the Cu, Cr, and C elements shown in the EDS spectrum belong to the measuring instrument itself.

Furthermore, the porous structure can reduce the effective permittivity of the material, which is advantageous to impedance matching [31, 32]. According to the subsequent analysis of the measured electromagnetic parameters of the samples, it is believed that this change may be beneficial to improve the degree of impedance matching of the samples, and then improve the electromagnetic wave absorption effect.

It is well known that the electromagnetic parameters of a material, including relative permittivity (*ε*_*γ*_*=ε′-jε″*) and relative complex permeability (*μ*_*γ*_*=μ′-jμ″*), play an extremely important role in the EMW absorption performance. The real part of the complex permittivity (*ε′* ) and the complex permeability (*μ′* ) show the storage capacity of the absorbing material for electrical and magnetic energy, while the imaginary part show loss capacity of electrical and magnetic energy [33, 34]. When these two electromagnetic parameters are close, this means that the material has a good impedance match. In this experiment, the electromagnetic parameters of samples were measured by dispersing the composites in a paraffin matrix with loading of 30wt% in the frequency range of 2–18 GHz. By substituting the measured electromagnetic parameters into the following formula, the reflection loss ability of the sample for an electromagnetic wave at different thicknesses can be simulated and calculated [35].

1 $$ {Z}_{\mathrm{in}}\kern0.5em =\kern0.5em {Z}_0\sqrt{\frac{\mu_{\gamma }}{\varepsilon_{\gamma }}}\tanh \left(\mathrm{j}\frac{2\pi \mathrm{fd}}{\mathrm{c}}\sqrt{\mu_{\gamma }{\varepsilon}_{\gamma }}\right) $$

2 $$ \mathrm{RL}\left(\mathrm{dB}\right)\kern0.5em =\kern0.5em 201\mathrm{og}\left|\frac{Z_{\mathrm{in}}\kern0.5em \hbox{-} \kern0.5em {Z}_0}{Z_{\mathrm{in}}\kern0.5em +\kern0.5em {Z}_0}\right| $$

Where ε′, ε″, μ′, and μ″ represent the real and imaginary parts of permittivity and permeability, respectively. The ƒ value is the frequency of electromagnetic wave, *d* is the thickness of the absorber, *Z*_0_ is the impedance of free space, Zin is the normalized input impedance, and *c* is the velocity of light in free space [36].

According to Formula (1)~(2), it can be concluded that when the reflection loss reaches − 20 dB, the corresponding material absorbs about 99% of the EMW, which means that the sample can be applied to actual needs [37].

**Fig. 4:**
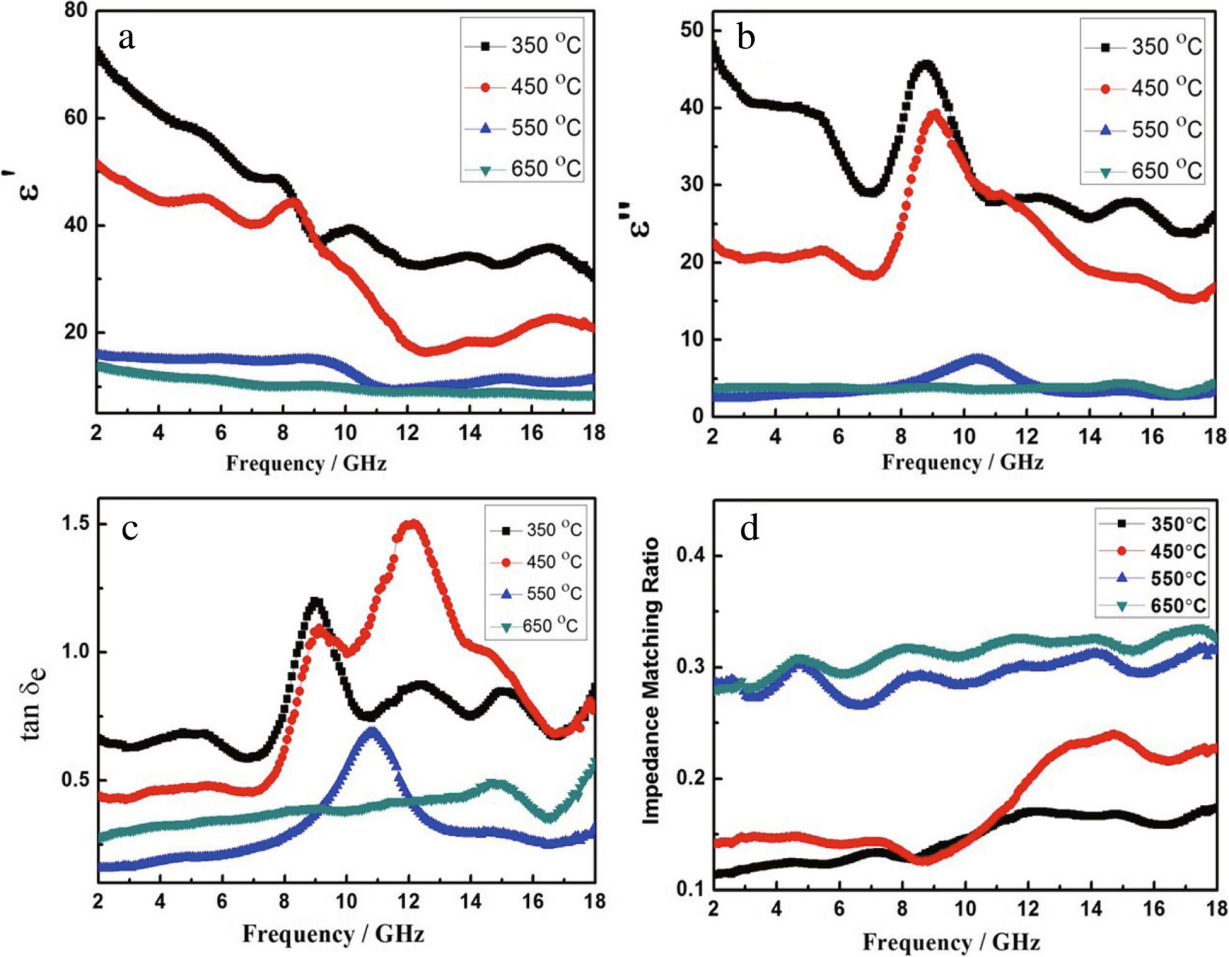
Frequency dependences of ε′ (**a**) and ε″ (**b**) of the mixtures with different calcining temperature. Dielectric loss factor (**c**) and impedance matching rate (**d**) of the samples by synthesized at different temperatures for frequency curves

The real part (ε′) and imaginary part (ε″) of the permittivity of the samples are shown in Fig. 4a, b, respectively, and the changes in the real and imaginary parts of the permittivity of the sample at different temperatures are carefully compared. It is shown that the value of ε′ decreases from 72.6 to 30.3 with increasing frequency for the sample with the annealing temperature of 350 °C. However, the ε″ value of the sample is showing different trends and the overall trend of decreasing in the test frequency range. There is a large fluctuation in the range of 7.1–10.4 GHz, which is mainly caused by dielectric relaxation. Obviously, the ε′and ε″ values of NiO/NiCo_2_O_4_ mixtures (550 °C and 650 °C) do not change significantly, compared with NiCo_2_O_4_ particles. It can be clearly seen from Fig. 3, the ε′ of the composites decreases as the annealed temperature increased. The electromagnetic parameters of the S3 and S4 have very similar trends and are different from the S1 and S2. In the frequency range of test, ε′ and ε″ of S3 and S4 varied in the range of 15.3 to 8.5 and 4.1 to 2.0, respectively. Based on the theory of free electrons, the high ε″ value of samples resulted in the high conductivity [38]. However, too high conductivity leads to the mismatching between permittivity and permeability, which is not favorable to the microwave absorption performance. When NiO crystal with higher electric resistivity is combined with NiCo_2_O_4_, the formation of electric conducting networks of the NiCo_2_O_4_ is prevented, thereby reducing the conductivity of the composites. For all samples, the *μ′* and μ″ of complex permeability in the whole frequency range, are very close to 1 and 0 even to negative, respectively [39, 40], (Additional file 1; Figure S1) which implied that the magnetism of samples is small and negligible.

In general, the reflection loss for electromagnetic wave material is related to the dielectric loss factor (tan*δ*_*e*_ *=* ε″|ε′). As shown in Fig. 4c, the dielectric loss factors for S3 and S4 are significantly smaller than for S1 and S2. The maximum dielectric loss factors for S3 and S4 are 0.69 (10.9 Hz) and 0.57 (18 Hz), respectively. The impedance matching ratio is widely used to demonstrate the dielectric loss ability of microwave absorbers [41]. The impedance matching ratio of the samples can be denoted as Eq. (3).

3 $$ {Z}_{\mathrm{r}}\kern0.5em =\kern0.5em \mid \frac{Z_{\mathrm{in}}}{Z_0}\mid \kern0.5em =\kern0.5em \mid \sqrt{\mu_{\gamma }/{\varepsilon}_{\gamma }}\tanh \left[j\left(2\pi \mathrm{fd}/\mathrm{c}\right)\sqrt{\mu_{\gamma }{\varepsilon}_{\gamma }}\right]\mid $$

In the Eq. (3), *f*, *c*, *Z*_in_, *Z*_0_, and *Z*_r_ is the attenuation constant, frequency, velocity of light, the input impedance of absorber, the impedance of free space, and the impedance matching ratio value, respectively. In order to further illustrate the electromagnetic loss properties of the samples, the impedance matching ration of the material is introduced and shown in Fig. 4d. Interestingly, we found that the impedance matching ratio of the NiO/NiCo_2_O_4_ mixtures is significantly higher than that of the S1 and S2. As a result, the former is more effective in absorbing electromagnetic wave.

**Fig. 5:**
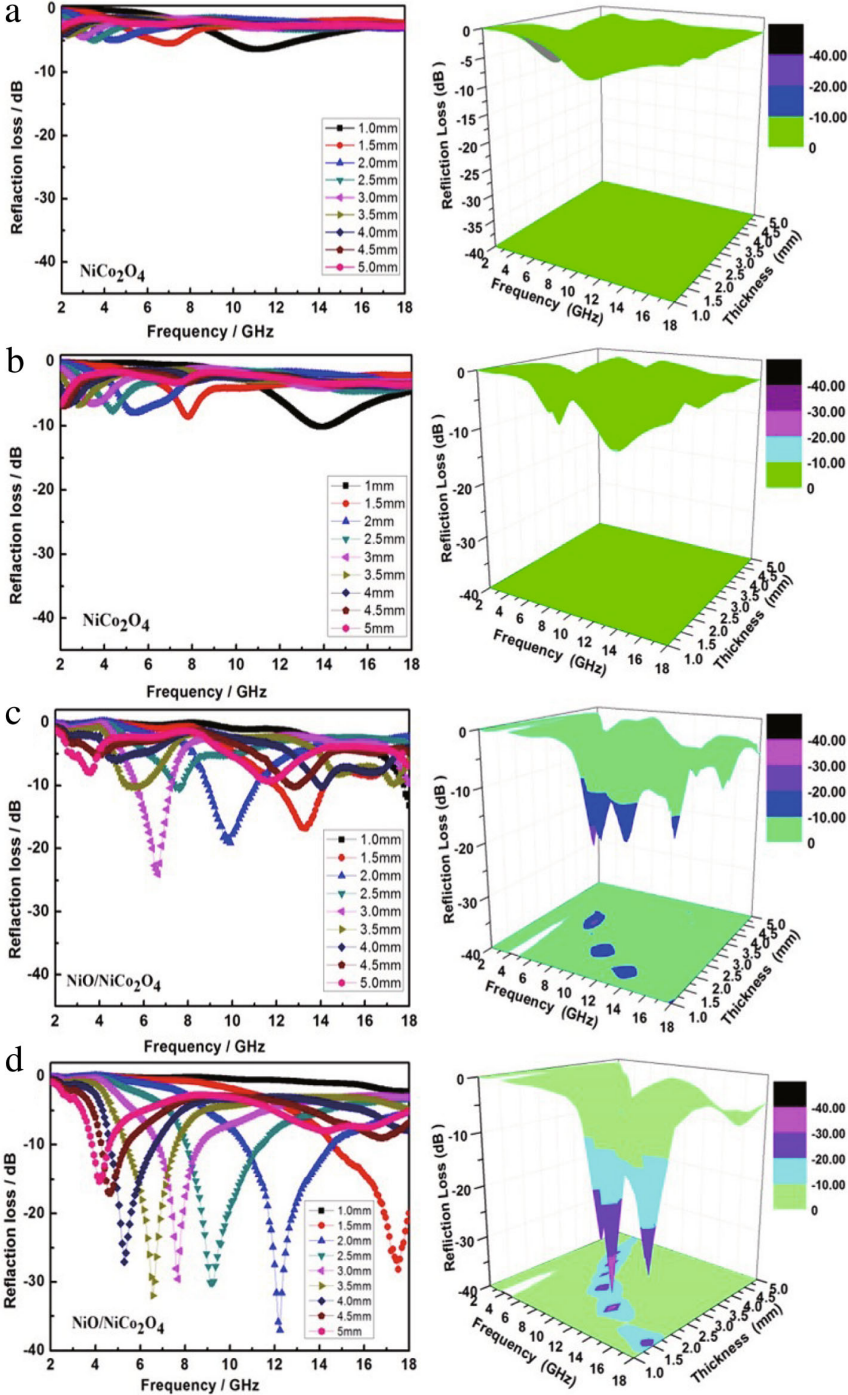
EM reflection loss curves of the samples. Where **a**–**d** represent the reflection loss curves of the samples with the annealing temperatures of 350 °C, 450 °C, 550 °C and 650 °C, and the images on the right correspond to the 3D reflection losses of the samples, respectively

It is obvious that the RL curve of samples can be used to reflect their microwave absorption performance. Based on the transmission line theory, it is possible to simulate and calculate the microwave absorbing parameters in the thickness range of 1.0–5.0 mm, according to the electromagnetic parameters. The theoretical RL curves of the samples calcinated at different temperatures in the frequency range of 2–18 GHz is shown in Fig. 5. It is generally considered that when the RL is lower than − 10 dB, the absorption rate of the electromagnetic wave of the sample can reach more than 90% [42], which is a typical performance index to be achieved by the application of the microwave absorption material. According to Fig. 5a and b, it is clearly indicated that the RL values of S1 and S2 are relatively poor and there is no bandwidth under − 10 dB. However, with the increase of NiO crystallinity in the samples, the minimum reflection loss of the NiO/NiCo_2_O_4_ mixtures is much lower than − 10 dB. Such as S4 shown in Fig. 5d, the range of frequency below − 10 dB correspond to the value of RL is 10.6~14.6 GHz and the bandwidth is 4.0 GHz. Meanwhile, we find that the minimum reflection loss reaches to − 37.0 dB at 12.2 GHz with the absorber thickness of 2.0 mm. In order to reflect the relationship between the RL and the thickness of the sample intuitively, the corresponding 3D contour curves are shown in Fig. 5. From the simulated electromagnetic wave reflection loss image, S4 would show excellent wave absorbing performance in the thickness range of 1.5–5.0 mm.

In addition to the inherent dipoles in NiCo_2_O_4_ and NiO phases, the defect dipoles are also generated due to the formation of lattice defects caused by phase transformation [28]. As a result, these dipoles would produce dielectric loss by orientation polarization relaxation in alternating electromagnetic fields. Interestingly, interfacial polarization relaxation will occur in NiO/NiCo_2_O_4_ mixtures with many heterogeneous interfaces, resulting in an enhanced dielectric loss. As shown in XRD pattern, when the annealing temperature reaches to 550 °C, some characteristic peaks such as 37.2°, 43.3°, and 62.9° can be found, which demonstrates the generation of NiO. The intensity of the diffraction peak of NiO at 35.49° is reinforced following the temperature, implying that more NiO crystals were produced.

**Fig. 6:**
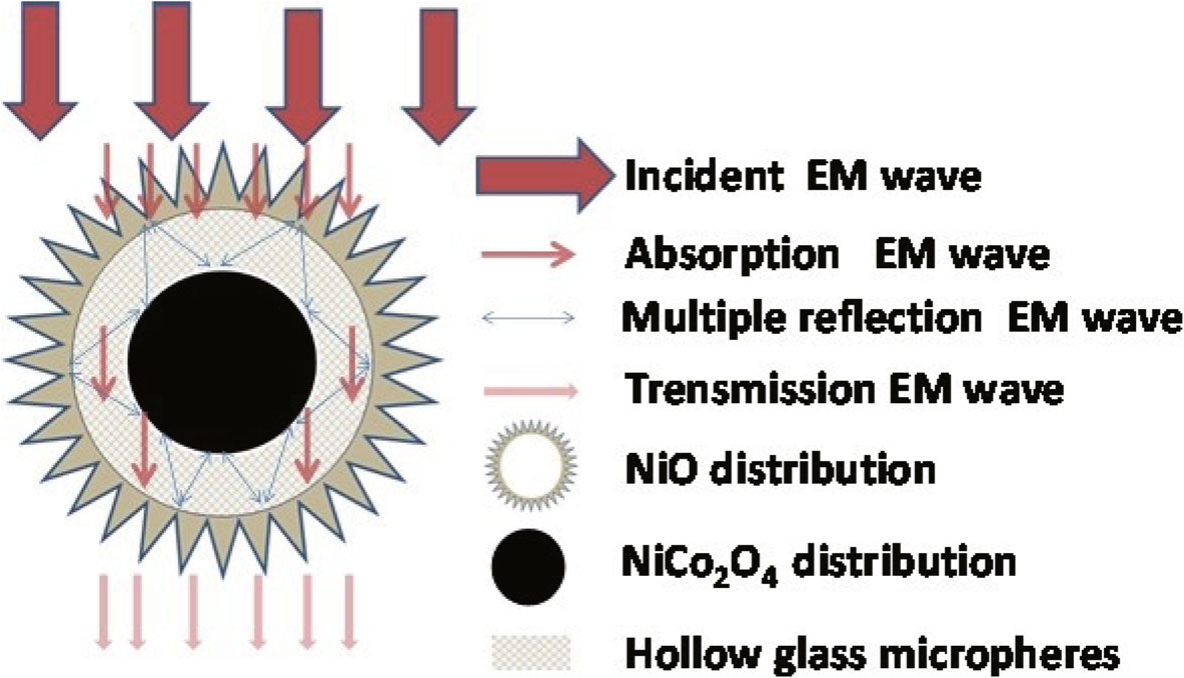
A schematic illustration of NiO/NiCo_2_O_4_ mixtures with yolk-shell structure to electromagnetic wave attenuation mechanism

In order to intuitionistic illustrate the possible mechanism, the diagrammatic map named Fig. 6 was provided. According to the figure, NiO/NiCo_2_O_4_ mixtures exhibit prominent microwave absorption property, which may be the following reasons. First, the NiO/NiCo_2_O_4_ mixtures have a rich heterogeneous interface, resulting in strong interfacial polarization relaxation, which leads to large dielectric losses. Secondly, the void space and interspaces in the shell-core structures enable the full exposure of NiO/NiCo_2_O_4_ mixture materials to the atmosphere, which facilitates the introduction of electromagnetic waves and produces dielectric resonance [43, 44]. Thirdly, the unique yolk-shell structure of NiO/NiCo_2_O_4_ mixtures can reflect and absorb the absorbed electromagnetic waves multiple times to enhance the loss of electromagnetic waves in the sample [45, 46].

## Conclusions

The NiO/NiCo_2_O_4_ mixtures with yolk-shell structure were prepared by hydrothermal method and followed by annealing at high temperature. When the annealing temperature is 650 °C, the NiO/NiCo_2_O_4_ mixtures exhibit the best microwave absorption properties, which is much better than the performance of pure NiCo_2_O_4_ and similar composites. The enhanced microwave absorption ability of the composites is mainly attributed to the interfacial polarization relaxation, orientation polarization relaxation caused by defect dipoles, and the unique yolk-shell structure. It is believed that such composites will be promising for widespread applications in the microwave absorption field.

## Additional file

Additional file 1: **Figure S1.** (a) Real part and (b) imaginary part of the complex permeability of the samples. (EPS 2207 kb)

## Abbreviations

EMW: Electromagnetic wave absorption;
RL: Reflection loss

## References

[1] Xiang J, Li J, Zhang X, Ye Q, Xu J, Shen X (2014). Magnetic carbon nanofibers containing uniformly dispersed Fe/Co/Ni nanoparticles as stable and high-performance electromagnetic wave absorbers. J Mater Chem A.

[2] Najim M, Modi G, Mishra YK, Adelung R, Singh D, Agarwala V (2015). Ultra-wide bandwidth with enhanced microwave absorption of electroless Ni–P coated tetrapod-shaped ZnO nano- and microstructures. Phys Chem Chem Phys.

[3] Liu J, Cao WQ, Jin HB, Yuan J, Zhang DQ, Cao MS (2015). Enhanced permittivity and multi-region microwave absorption of nanoneedle-like ZnO in the X-band at elevated temperature. J Mater Chem C.

[4] Zhang X, Yan F, Zhang S, Yuan H, Zhu C, Zhang X, Chen Y (2018). Hollow N-doped carbon polyhedron containing CoNi alloy nanoparticles embedded within few-layer N-doped graphene as high-performance electromagnetic wave absorbing material. ACS Appl Mater Interfaces.

[5] Liu W, Liu L, Yang Z, Xu J, Hou Y, Ji G (2018). A versatile route toward the electromagnetic functionalization of metal–organic framework-derived three-dimensional nanoporous carbon composites. ACS Appl Mater Interfaces.

[6] Wu H, Wang L, Wang Y, Guo S, Shen Z (2012). Enhanced microwave performance of highly ordered mesoporous carbon coated by Ni_2_O_3_ nanoparticles. J Alloys Compd.

[7] Yuan H, Zhang X, Yan F, Zhang S, Zhu C, Li C, Zhang X, Chen Y (2018). Nitrogen-doped carbon nanosheets containing Fe_3_C nanoparticles encapsulated in nitrogen-doped graphene shells for high-performance electromagnetic wave absorbing materials. Carbon.

[8] Liu X, Cui X, Chen Y, Zhang XJ, Yu R, Wang GS, Ma H (2015). Modulation of electromagnetic wave absorption by carbon shell thickness in carbon encapsulated magnetite nanospindles–poly(vinylidene fluoride) composites. Carbon.

[9] Ding Y, Zhang L, Liao Q, Zhang G, Liu S, Zhang Y (2016). Electromagnetic wave absorption in reduced graphene oxide functionalized with Fe_3_O_4_/Fe nanorings. Nano Res.

[10] Liu Jiu Rong, Itoh Masahiro, Machida Ken-ichi (2006). Magnetic and electromagnetic wave absorption properties of α-Fe∕Z-type Ba-ferrite nanocomposites. Applied Physics Letters.

[11] Qu B, Zhu C, Li C, Zhang X, Chen Y (2016). Coupling hollow Fe_3_O_4_-Fe nanoparticles with graphene sheets for high-performance electromagnetic wave absorbing material. ACS Appl Mater Interfaces.

[12] Wang Y, Wang W, Zhu M, Yu D (2017). Electromagnetic wave absorption polyimide fabric prepared by coating with core–shell NiFe_2_O_4_@PANI nanoparticles. RSC Adv.

[13] Liu W, Shao Q, Ji G, Liang X, Cheng Y, Quan B, Du Y (2017). Metal–organic-frameworks derived porous carbon-wrapped Ni composites with optimized impedance matching as excellent lightweight electromagnetic wave absorber. Chem Eng J.

[14] Zhang X, Ji G, Liu W, Zhang X, Gao Q, Li Y, Du Y (2016). A novel Co/TiO_2_ nanocomposite derived from a metal–organic framework: synthesis and efficient microwave absorption. J Mater Chem C.

[15] Liu P, Huang Y, Yan J, Zhao Y (2016). Magnetic graphene@PANI@porous TiO_2_ ternary composites for high-performance electromagnetic wave absorption. J Mater Chem C.

[16] Liu Y, Chen Z, Zhang Y, Feng R, Chen X, Xiong C, Dong L (2018). Broadband and lightweight microwave absorber constructed by in situ growth of hierarchical CoFe_2_O_4_/reduced graphene oxide porous nanocomposites. ACS Appl Mater Interfaces.

[17] Moitra D, Dhole S, Ghosh BK, Chandel M, Jani RK, Patra MK, Vadera SR, Ghosh NN (2017). Synthesis and microwave absorption properties of BiFeO_3_ nanowire-RGO nanocomposite and first-principles calculations for insight of electromagnetic properties and electronic structures. J Phys Chem C.

[18] Yan F, Guo D, Zhang S, Li C, Zhu C, Zhang X, Chen Y (2018). An ultra-small NiFe_2_O_4_ hollow particle/graphene hybrid: fabrication and electromagnetic wave absorption property. Nanoscale.

[19] Yan F, Zhang S, Zhang X, Li C, Zhu C, Zhang X, Chen Y (2018). Growth of CoFe_2_O_4_ hollow nanoparticles on graphene sheets for high-performance electromagnetic wave absorbers. J Mater Chem C.

[20] Yang R, Wang B, Xiang J, Mu C, Zhang C, Wen F, Wang C, Su C, Liu Z (2017). Fabrication of NiCo_2_-anchored graphene nanosheets by liquid-phase exfoliation for excellent microwave absorbers. ACS Appl Mater Interfaces.

[21] Yuan H, Yan F, Li C, Zhu C, Zhang X, Chen Y (2017). Nickel nanoparticle encapsulated in few-layer nitrogen-doped graphene supported by nitrogen-doped graphite sheets as a high-performance electromagnetic wave absorbing material. ACS Appl Mater Interfaces.

[22] Hu L, Wu L, Liao M, Hu X, Fang X (2012). Electrical transport properties of large, individual NiCo_2_O_4_ nanoplates. Adv Funct Mater.

[23] Zhan J, Yao Y, Zhang C, Li C (2014). Synthesis and microwave absorbing properties of quasione-dimensional mesoporous NiCo_2_O_4_ nanostructure. J Alloys Compd.

[24] Zhou M, Lu F, Chen B, Zhu X, Shen X, Xia W, He H, Zeng X (2015). Thickness dependent complex permittivity and microwave absorption of NiCo_2_O_4_ nanoflakes. Mater Lett.

[25] Luo H, Wang X, Song K, Yang J, Gong R (2016). Enhanced microwave absorption properties of flexible polymer composite based on hexagonal NiCo_2_O_4_ microplates and PVDF. J Electron Mater.

[26] Wang L, Xing H, Gao S, Ji X, Shen Z (2017). Porous flower-like NiO@graphene composites with superior microwave absorption properties. J Mater Chem C.

[27] Wang Y, Wu X, Zhang W, Li J, Luo C, Wang Q (2017). Fabrication and enhanced electromagnetic wave absorption properties of sandwich-like graphene@NiO@PANI decorated with Ag particles. Synth Met.

[28] Liu X, Hao C, Jiang H, Zeng M, Yu R (2017). Hierarchical NiCo_2_O_4_/Co_3_O_4_/NiO porous composite: a lightweight electromagnetic wave absorber with tunable absorbing performance. J Mater Chem C.

[29] Liang X, Quan B, Chen J, Tang D, Zhang B, Ji G (2017). Strong electric wave response derived from the hybrid of lotus roots-like composites with tunable permittivity. Sci Rep.

[30] Liu P, Yang M, Zhou S, Huang Y, Zhu Y (2019). Hierarchical shell-core structures of concave spherical NiO nanospines@carbon for high performance supercapacitor electrodes. Electrochim Acta.

[31] Kim IH, Lee H, Yu A, Jeong JH, Lee Y, Kim MH, Lee C, Kim YD (2018). Synthesis and catalytic activity of electrospun NiO/NiCo_2_O_4_ nanotubes for CO and acetaldehyde oxidation. Nanotechnology.

[32] Liu Qinglei, Zhang Di, Fan Tongxiang (2008). Electromagnetic wave absorption properties of porous carbon/Co nanocomposites. Applied Physics Letters.

[33] Du Y, Liu W, Qiang R, Wang Y, Han X, Ma J, Xu P (2014). Shell thickness-dependent microwave absorption of core-shell Fe_3_O_4_@C composites. ACS Appl Mater Interfaces.

[34] Qiang R, Du Y, Zhao H, Wang Y, Tian C, Li Z, Han X, Xu P (2015). Metal organic framework-derived Fe/C nanocubes toward efficient microwave absorption. J Mater Chem A.

[35] Che RC, Peng LM, Duan XF, Chen Q, Liang XL (2004). Microwave absorption enhancement and complex permittivity and permeability of Fe encapsulated within carbon nanotubes. Adv Mater.

[36] Du L, Du YC, Li Y, Wang JY, Wang C, Wang XH, Xu P, Han XJ (2010). Surfactant-assisted solvothermal synthesis of Ba(CoTi)_x_Fe_12-2x_O_19_ nanoparticles and enhancement in microwave absorption properties of polyaniline. J Phys Chem C.

[37] Wang Fenglong, Liu Jiurong, Kong Jing, Zhang Zijun, Wang Xinzhen, Itoh Masahiro, Machida Ken-ichi (2011). Template free synthesis and electromagnetic wave absorption properties of monodispersed hollow magnetite nano-spheres. Journal of Materials Chemistry.

[38] Wei S, Wang X, Zhang B, Yu M, Zheng Y, Wang Y, Liu J (2017). Preparation of hierarchical core-shell C@NiCo_2_O_4_@Fe_3_O_4_ composites for enhanced microwave absorption performance. Chem Eng J.

[39] Zhang X. F., Guan P. F., Dong X. L. (2010). Transform between the permeability and permittivity in the close-packed Ni nanoparticles. Applied Physics Letters.

[40] Liu Y, Xing H, Wang L, Liu Z, Wang H, Jia H (2018). Novel microwave absorption materials of porous flower-like nickel oxide@polyaniline in the X-band. Nano.

[41] Lv H, Ji G, Liang X, Zhang H, Du Y (2015). A novel rod-like MnO_2_@Fe loading on graphene giving excellent electromagnetic absorption properties. J Mater Chem C.

[42] Zhang H, Tian X, Wang C, Luo H, Hu J, Shen Y, Xie A (2014). Facile synthesis of RGO/NiO composites and their excellent electromagnetic wave absorption properties. Appl Surf Sci.

[43] Zhang L, Zhang X, Zhang G, Zhang Z, Liu S, Li P, Liao Q, Zhao Y, Zhang Y (2015). Investigation on the optimization, design and microwave absorption properties of reduced graphene oxide/tetrapod-like ZnO composites. RSC Adv.

[44] Liu QC, Zi ZF, Zhang M, Pang AB, Dai JM, Sun YP (2014). Enhanced microwave absorption properties of urchin-like Fe/α-Fe_2_O_3_ composite synthesized by a simple thermal oxidation. Integr Ferroelectr.

[45] Liu Q, Zi Z, Zhang M, Zhang P, Pang A, Dai J, Sun Y (2013). Solvothermal synthesis of hollow glass microspheres/Fe_3_O_4_ composites as a lightweight microwave absorber. J Mater Sci.

[46] Quan B, Liang X, Ji G, Ma J, Ouyang P, Gong H, Xu G, Du Y (2017). Strong electromagnetic wave response derived from the construction of dielectric/magnetic media heterostructure and multiple interfaces. ACS Appl Mater Interfaces.

